# Student perceptions of evaluation in undergraduate medical education: A qualitative study from one medical school

**DOI:** 10.1186/1472-6920-12-45

**Published:** 2012-06-22

**Authors:** Sarah Schiekirka, Deborah Reinhardt, Susanne Heim, Götz Fabry, Tobias Pukrop, Sven Anders, Tobias Raupach

**Affiliations:** 1 Department of Cardiology and Pneumology, University Hospital Göttingen, D-37099, Göttingen, Germany; 2 Department of Medical Psychology and Sociology, Georg-August University Göttingen, Göttingen, Germany; 3 Department of Medical Psychology, Albert-Ludwigs-University Freiburg, Freiburg, Germany; 4 Department of Haematology and Oncology, University Hospital Göttingen, Göttingen, Germany; 5 Department of Legal Medicine, University Medical Centre Hamburg Eppendorf, Hamburg, Germany

**Keywords:** Undergraduate medical education, Evaluation, Purpose, Consequence, Learning outcome

## Abstract

**Background:**

Evaluation is an integral part of medical education. Despite a wide use of various evaluation tools, little is known about student perceptions regarding the purpose and desired consequences of evaluation. Such knowledge is important to facilitate interpretation of evaluation results. The aims of this study were to elicit student views on the purpose of evaluation, indicators of teaching quality, evaluation tools and possible consequences drawn from evaluation data.

**Methods:**

This qualitative study involved 17 undergraduate medical students in Years 3 and 4 participating in 3 focus group interviews. Content analysis was conducted by two different researchers.

**Results:**

Evaluation was viewed as a means to facilitate improvements within medical education. Teaching quality was believed to be dependent on content, process, teacher and student characteristics as well as learning outcome, with an emphasis on the latter. Students preferred online evaluations over paper-and-pencil forms and suggested circulating results among all faculty and students. Students strongly favoured the allocation of rewards and incentives for good teaching to individual teachers.

**Conclusions:**

In addition to assessing structural aspects of teaching, evaluation tools need to adequately address learning outcome. The use of reliable and valid evaluation methods is a prerequisite for resource allocation to individual teachers based on evaluation results.

## Background

Programme evaluation in medical education should be multi-dimensional, combining subjective and objective data to gather comprehensive information on teaching processes and learning outcomes [1,2]. Scaled ratings provided by students are widely used to evaluate courses and teachers despite a large body of literature questioning the reliability and validity of this approach [3,4]. In fact, many traditional evaluation forms using these scales assess ‘teaching quality’ in terms of student satisfaction with courses and organisational/structural aspects of teaching rather than the actual increase of knowledge or skills [5,6]. Using these surrogate parameters to appraise teaching quality as a whole can be misleading as student ratings might be biased by the initial interest of students [7], instructor reputation [8] and instructor enthusiasm [9,10]. Most studies addressing these confounders were quantitative in nature and did not allow any conclusions to be drawn on the decision-making process underlying the critical appraisal of courses and teachers by students.

Few qualitative studies have focused on student perceptions of course evaluation and processes potentially affecting numeric results. In one of these studies, Billings-Gagliardi et al. [11] asked medical students to think aloud while completing a typical basic science course evaluation. Findings indicated that judgements were partially based on unique or unexpected criteria, and thereby questioned fundamental assumptions frequently underlying the interpretation of evaluation results. At the very least, medical educators need to be aware that students completing evaluation forms may be guided by different priorities than programme directors interpreting evaluation results. Faculty should know how students perceive the goals and consequences of evaluations, and any other factors that might influence ratings. Understanding how students view evaluation, define good teaching and arrive at course ratings is of utmost importance; evaluation results currently guide resource-allocation within medical schools [2]. We are concerned that this current use of data derived from traditional evaluation forms (i.e. mainly using scaled questions) fails to acknowledge the impact that student perceptions of evaluation may have on evaluation results.

In order to further elucidate student attitudes towards course evaluation, focus group interviews were conducted, addressing student perceptions of evaluation goals, use of evaluation tools, individual benchmarks for teaching quality and the possible consequences of the evaluation results. We hypothesised that students would be aware of the multiple dimensions of course evaluation and that interviews might provide an insight into processes underlying the completion of traditional evaluation forms.

## Methods

### Course evaluation at Göttingen medical school

The six-year undergraduate medical curriculum comprises two pre-clinical and three clinical years, followed by a practice year. The clinical part of the curriculum adopts a modular structure with 21 modules of different length occurring in a fixed order over three years. At the end of each teaching module, students are invited to complete online evaluation forms containing one overall module rating and five questions assessing specific aspects. These questions address the implementation of interdisciplinary teaching, promotion of self-directed learning, perceived learning outcome in relation to future job requirements, structure of a module and practical aspects such as cancellation of teaching sessions. Students are invited to rate all aspects on six-point scales. In order to increase the range of aspects covered in course evaluation, a novel evaluation tool addressing actual learning outcome [12] was recently added to the pre-existing evaluation system at our institution. Students were not completely familiar with the novel tool, thus this study focused on student perceptions of the traditional evaluation tool of our institution.

### Focus group interviews

All medical students in Years 3 and 4 of undergraduate education were contacted by e-mail and invited to participate in focus group interviews addressing general attitudes towards course evaluation as well as issues related to current evaluation processes at our institution. We only included students from the clinical phase of training as evaluation practices are different and less standardised in pre-clinical years. However, we did include students at different stages of the clinical curriculum – i.e., those in the first, third and fourth out of six half-year terms – in order to increase the representativeness of the sample. During summer 2011, three separate focus group sessions including five to seven students each (N = 17; 13 female and 4 male) were conducted [13]. Sessions were moderated by one of the authors (SS). For compensation, every student received a book voucher worth € 25. Discussions lasted between 59 and 75 minutes. We used open-ended questions focusing on student perceptions of teaching quality and programme evaluation in general. In order to ensure consistency across groups, the following trigger questions were used to guide the interviews:

· In your opinion, what is the purpose of evaluation in medical education?

· How would you define good teaching?

· What do you think about the evaluation tools currently used at our institution?

· How do you arrive at an overall course rating?

· What kind of consequences would you like to see to be drawn from course evaluations?

### Data analysis

Focus group sessions were audio-taped and transcribed verbatim. Subsequently, two independent investigators (SS & DR) categorised individual contributions to the discussion based on qualitative content analysis [14] using the MaxQDA software (VERBI GmbH, Marburg, Germany). The trigger questions served as orientation for coding, and subthemes were identified in an iterative process, which ensured that themes were comparable across groups. Themes and subthemes that emerged from each group were subsequently included in mind maps, which generated four overarching themes (see Results). Findings from the third focus group did not add substantially to existing themes; thus, theoretical saturation was reached [13], and no further sessions were organised.

The local Institutional Review Board (application number 27/3/11) waived ethics approval as the study protocol was not deemed to represent bio-medical or epidemiological research. Procedures complied with data protection rules, and all data were anonymised prior to analysis.

## Results

In accordance with the trigger questions listed above, four distinct themes emerged during focus group discussions. These were related to teaching quality, perceptions of evaluation, evaluation tools and data collection, and consequences that might be drawn on the basis of evaluation results. Main aspects arising during discussions are summarised in Table 1.

**Table 1:** Main aspects arising during discussions (sorted by category); see text for details

**Indicators of high quality teaching**	**Perceptions of evaluation**	**Evaluation tools** **and data collection**	**Consequences of evaluation**
Content ·alignment of teaching to student level ·prioritisation of important aspects ·no inadequate redundancy ·coverage of most relevant content in examinations	Goals ·improve teaching processes and their outcome ·provide individual feedback to teachers ·assess whether learning objectives have been met	Evaluation targets ·learning outcome ·procedural and structural aspects of teaching (time-tables, facilities etc.) ·adequacy of examinations ·individual teacher performance	Publication of results ·free access to all faculty ·course rankings ·protection of individual data ·discussion of delicate data in ‘evaluation committees’
Process ·preponderance of interactive teaching formats ·free access to teaching materials ·frequent teacher feedback on student performance	Barriers against participation ·too frequent evaluations ·lack of feedback ·lack of time specifically dedicated to evaluation	Preferred format ·open (vs. scaled) questions ·no more than 15 items ·online (vs. paper and pencil) ·voluntary participation	Rewards for positive results ·monetary rewards ·extra time off
Teacher characteristics ·subject knowledge ·clinical experience ·good preparation ·enthusiasm	Confounders ·individual definitions of “good” teaching ·student motivation ·positive attitude/motivation ·halo effects	Preferred time-point ·after end-of-course examinations ·‘continuous’ evaluation (online platform)	Consequences of negative results ·compulsory teacher training ·exemption from teaching activities
Learning outcome ·not restricted to specific subjects ·preparation for life-long learning			Feedback loop ·disclosure of actions taken in response to evaluation results

### Teaching quality (trigger question: “How would you define good teaching?”)

According to students, teaching quality was dependent on content, process (including examinations), teacher and student characteristics as well as learning outcome.

Regarding content, students felt that teaching should be aligned to their current level of knowledge and skills. Topics should be prioritised according to clinical/practical relevance and weighted as proposed by the learning objectives of the institution. Notwithstanding the need for a reasonable amount of repetition, excessive redundancy should be avoided by negotiating content between modules. Finally, high congruence between the content taught and the content covered in end-of-course examinations was perceived as important.

"“To me, good teaching is something that can be transferred to clinical practice.” (Year 4, male student)"

As far as procedural aspects of teaching were concerned, students preferred interactive teaching over traditional didactic lectures and wanted free and easy access to teaching materials. In addition, they valued teacher feedback on their practical skills.

"“I think that interactive, small-group teaching is the best way to teach and learn.” (Year 4, female student)"

Students preferred dedicated teachers who view educational activities as an integral part of their professional role. Ideally, teachers should be knowledgeable, clinically experienced, well-prepared and enthusiastic. The role of teacher training was discussed, and some students doubted that current training programmes were effective for teachers with low motivation to engage in medical education.

"“Teaching completely hinges on the teacher.” (Year 4, female student)"

In addition to being fun, participation in teaching sessions should result in manifest learning outcomes which are not restricted to the content area of a specific subject but also encompass general skills such as life-long learning. Students were well aware of their own responsibility for achieving favourable learning outcomes, and acknowledged the need to prepare for courses and the importance of their own motivation in achieving a desired outcome.

"“My own learning outcome is crucial to me.” (Year 3, female student)"

A number of comments addressed the weight attributed to teaching as compared with research activities within medical schools. Students felt that teaching was not perceived as a priority by many physicians and asked for consultants to prioritise teaching and promote this attitude within their departments.

"“A professor of casual surgery has to do and publish good research, needs to have patients who are satisfied with his work and spread the word – and teaching comes last. He does not get any extra money, external funds or great renown – he gets nothing for teaching.” (Year 4, female student)"

### Perceptions of evaluation (Trigger questions: “In your opinion, what is the purpose of evaluation in medical education?” and “How do you arrive at an overall course rating?”)

This section summarises perceptions of the purpose of course evaluation as well as the approach of the student towards completing evaluation forms. A number of statements indicated that the overall goal of evaluation was to improve teaching processes and their outcome. Students felt that participation in the evaluation process enabled them to express their views on courses. In addition to providing specific feedback to teachers, evaluation was considered a means to assess whether learning objectives had been met in a specific course.

"“In general, I would give a positive rating for courses in which I got the feeling to have learned a lot in a pleasant manner.” (Year 3, female student)"

Evaluation activities that occurred during lectures were perceived as distracting, and some students were confused by the large number of evaluation forms they were asked to complete. Another barrier to participation was a lack of feedback regarding the possible consequences of the evaluation results.

Students acknowledged that individual preferences and definitions of ‘good’ teaching may considerably impact overall course ratings. According to some comments, global ratings were difficult to generate and dependent on gut feelings, i.e. whether they liked and were motivated by a course. In addition, perceived quality and difficulty of end-of-course examinations were likely to mediate a halo effect on overall course ratings.

"“If something really annoys me about a particular module, my overall rating will be generally lower (…) Even if I was not happy with 10% of the module and the rest was OK, I will give a lower rating since the bad aspects tend to linger in my memory.” (Year 4, male student)"

### Evaluation tools and data collection (trigger question: “What do you think about the evaluation tools currently used at our institution?”)

Comments regarding evaluation tools and methods of data collection were related to targets of the evaluation process and preferred question formats, as well as the frequency and practical aspects of evaluation.

According to students, evaluation tools should capture actual learning outcome and judge procedural and organisational aspects of teaching. The adequacy of examination questions and their relation to course content were also suggested as evaluation targets. Finally, emphasis was placed on individual and specific evaluation of teachers.

"“I think, as a teacher, if I received a ‘3’ rating from all students – how am I supposed to make sense of that?’ However, if the comment read ‘good overall but – whatever – presentation slide design was not ideal’ that would be a particular point I could try to improve on.” (Year 3, female student)"

With respect to the format of evaluation tools, students preferred open questions on evaluation forms as well as interactive discussions with module representatives. Scaled questions received considerably less support as they were not believed to provide useful information.

"“Overall ratings may be easy to analyse statistically but I don’t think they really tell you anything.” (Year 3, female student)"

Students suggested a maximum of 15 questions on any single evaluation form. Online evaluations were preferred over paper-and-pencil forms although students admitted to postponing or forgetting the completion of online evaluations as they were not given high priority. Students were unsure about the ideal time-point of evaluation, but many favoured completion of forms following end-of-course examinations. Others suggested providing constant access to an online platform in order to be able to enter any comments as they emerged. This was consistent with a general claim for evaluation tools to be simple and easy to use. In addition, most students agreed that participation in course evaluation should be voluntary. At the same time, they acknowledged that minimum response rates are needed to obtain reliable and valid results. Comments on how evaluation results might be used to improve teaching are described in the following section.

### Proposed consequences of course evaluation (trigger question: “What kind of consequences would you like to see to be drawn from course evaluations?”)

Regarding the handling of evaluation results, students suggested all data be published within their medical school; some felt that official course rankings could be used as motivators. However, students also acknowledged the need to protect individual teachers’ data. One option to resolve this could be to discuss individual evaluation results with teachers in a protected environment (e.g., in an ‘evaluation committee’).

The majority of comments addressed feedback for individual teachers and possible consequences of positive or negative ratings. Students felt that the principal goal of providing individual feedback was to facilitate improvement in teaching skills; therefore, free text comments were preferred over scaled ratings. Individual characteristics and a lack of motivation were mentioned as potential barriers against changing individual teaching behaviour.

"“If someone is simply not interested in teaching, nothing is going to change at all because his job is safe – he’s just not interested.” (Year 4, male student)"

When asked about potential consequences for individual teachers, students generally preferred incentives over punishments. There was some debate on the ideal type of incentives with half of the students favouring extra time off and others suggesting monetary rewards for good teachers. There was also disagreement regarding the approach to teachers with particularly negative evaluation results. One option would be to implement a compulsory training programme. Alternatively, bad teachers could be excluded from teaching activities. However, as physicians working in medical school hospitals are obliged to teach even if they do not like this part of their job, exemption from teaching duties based on negative evaluations may even be interpreted as a reward.

"“If I take someone who is definitely not up for teaching, I will never motivate him to deliver good teaching – so maybe the whole system behind it needs to be changed slightly.” (Year 4, female student)"

A considerable number of students stated that they wished to receive feedback on the consequences drawn from evaluation results:

"“I believe that more students would be willing to evaluate if they knew that it is of some avail.” (Year 4, female student)"

## Discussion

This is one of the first qualitative studies of student perceptions of evaluation in undergraduate medical education. Our results might be of interest to faculty and programme directors who need to be aware of the assumptions and confounders underlying student ratings. This is of particular importance if evaluation results are used to guide resource allocation within medical schools [15]. Medical students participating in focus group interviews identified almost all relevant aspects of course evaluation reported in the literature [16,17] and were aware that this institutional process should gauge teaching quality by addressing various areas such as the content taught, teacher characteristics, and – most importantly – learning outcome. However, a number of contributions revealed that students did not use specific pre-defined criteria of good teaching (i.e., ‘benchmarks’) when appraising teaching quality. In the absence of such criteria, students referred to their gut feeling and single outstanding (negative or positive) events as major contributors to their overall course ratings. As many students preferred evaluation activities to be scheduled after end-of-course examinations, subjective ratings of teaching quality including learning outcome might be confounded by examination difficulty and individual scores [18]. Unfortunately, a recent study on end-of-course examinations doubted that international minimum standards of assessment quality are currently being maintained in German medical schools, thus questioning the validity of exam scores regarding actual learning outcome [19]. In addition, the definition of a successful individual learning outcome might substantially differ between students and medical educators. For example, a number of German Associate Deans for Medical Education have proposed to judge teaching success based on *aggregated* examination scores and pass rates [2], while the students interviewed in this study were mainly interested in *individual* learning outcome.

Our finding of a wide variety of confounders affecting student ratings is in line with previous quantitative research [7,8] and suggests that overall course ratings may reflect student satisfaction with courses and teachers rather than teaching quality or actual learning outcome [20]. Obviously, satisfaction with teaching is likely to result in higher motivation to learn, thus rendering student satisfaction an important moderator of learning behaviour and, eventually, learning success. However, faculty need to be aware that traditional evaluation tools do not explicitly measure outcome. We recently reported on a novel evaluation tool aimed at determining learning outcome regarding specific learning objectives [12]. By using repetitive student self-assessments to calculate performance gain for specific learning objectives from all domains of medical education (knowledge, skills and attitudes), this tool produced reliable and valid data in one pilot study. In addition, results obtained with this outcome-based tool appeared to be unrelated to overall course ratings provided by students, thus potentially adding a new dimension to traditional evaluation tools [4]. Obviously, this method should not replace evaluation focussing on structural and procedural aspects of teaching. Instead, it may add value to existing evaluation systems [1].

Students enumerated several quality indicators of teaching that encompassed a wide range of parameters pertaining to teachers, courses and the medical school as a whole. In contrast, suggestions regarding consequences to be drawn from evaluation results were mainly directed at individual teachers. This may be due to the fact that teacher characteristics appear to be crucial for student perceptions of teaching quality. While more research into this issue is warranted [21], it may be hypothesised that empathic and enthusiastic teachers can improve student learning by increasing their motivation to learn. However, this aspect is rarely specifically addressed in evaluation forms. Given the importance of the individual teacher, it does seem natural for students to favour evaluation systems entailing direct consequences for specific teachers. Most students preferred incentives over negative consequences. Published reports of instruments to increase motivation to teach usually refer to positive reinforcement measures [22-24]. In order to distinguish effective from potentially detrimental incentive systems, the views of teachers and programme directors should be considered. To this end, focus group discussions involving these stakeholders of undergraduate medical education may be useful.

Students listed a number of course and teacher characteristics that are frequently addressed in faculty development programmes (i.e., alignment of teaching to student level, prioritisation of important content, teacher feedback, adequacy of examinations; see Table 1) [25]. This list stresses the relevance of teacher training with regard to improving teaching quality and increasing student motivation to learn. However, students were ambivalent regarding the effectiveness of teacher training in individuals with low motivation to teach.

As far as evaluation format was concerned, students consistently preferred online evaluations over paper-and-pencil methods. At the same time, participation in online evaluations was not given high priority, and students tended to postpone or forget to log on. Low response rates have been reported by many institutions using online methods; there is currently no clear solution to this problem [26,27]. There was a general concern that evaluation frequently fails to meet its primary goal of improving teaching quality [28]. These concerns might be addressed by providing students with feedback on the consequences of evaluation.

### Limitations and suggestions for further research

Focus group discussions are a useful adjunct to quantitative statistical methods [29-31]. However, they have certain limitations. Thus, while providing in depth information on individual opinions and specific problems, they may not be fully representative of the group of interest. Both the number of groups and the number of students included were small but within the range used in similar research [32]. Group composition was similar for all groups, and we did not attempt to sample specific sub-groups. Discussions were focussed on the issue of evaluation, and interviews were standardised [31]. As no major new themes emerged from the third group discussion, it is likely that sampling was adequate for current purposes.

Only students voluntarily signing up for focus group discussions were included in the study. Thus, potential self-selection bias might have favoured those particularly interested in the subject. The proportion of female participants in focus groups (76%) was similar to the percentage (65%) recently found in a nationwide survey of German medical students [33]. Since gender does not appear to impact heavily on evaluation results [21], the slight over-representation of females in our sample is unlikely to threaten the validity of our findings.

Rather than producing statistically representative data, qualitative research facilitates easy identification of general trends or patterns regarding the attitudes of the target group, establishing ‘functional and psychological representativeness’ [34]. However, the assumption that data collection was relatively comprehensive is supported by the identification of a large number of aspects known to be relevant from more representative research (see above).

Moderators or participants themselves may influence the behaviour and responses of discussants. We have no reason to assume that our results have been particularly confounded by such factors; however, we cannot rule out this bias as a potential limitation of our study. To date, very few qualitative studies have focussed on student perceptions of evaluation. As a consequence, the validity of our findings needs to be confirmed in further studies in order to assess the generalizability of our results to other institutions and study subjects. While this study generated a set of variables deemed important by students, quantitative studies are needed to estimate the actual impact each of these factors has on student ratings of teaching quality. Finally, future research should be directed at the perspectives of teachers and programme directors on evaluation.

## Conclusion

In addition to procedural and structural aspects of teaching, learning outcome was viewed as an important target for evaluation. Accordingly, evaluation tools need to adequately address learning outcome. Proposed consequences to be drawn from evaluation results were mainly directed at individual teachers rather than institutions or teaching modules. Evaluation methods must be reliable and valid in order to be used as the basis for resource allocation to individual teachers.

## Competing interests

None of the authors has any conflict of interest to declare.

## Authors’ contributions

SS helped to design the study, conducted focus group interviews, prepared data analysis and contributed to the manuscript draft. DR transcribed focus group interviews and contributed to data analysis as well as manuscript drafting. SH and GF helped to design the study, gave methodological advice, contributed to data interpretation and were both involved in manuscript preparation. TP and SA provided advice on methodology and contributed to the discussion. TR conceived of the study, was involved in the design of the study, analysed the data and wrote the manuscript. All authors read and approved the final manuscript.

## Authors’ information

SARAH SCHIEKIRKA is a psychologist at Göttingen University Hospital. She is primarily involved in higher education research, especially clinical teaching and evaluation.

DEBORAH REINHARDT is a medical student at Göttingen University. She is currently preparing her doctoral thesis on course evaluation.

SUSANNE HEIM is a social scientist working in the Department of General Practice at Göttingen University. She conducted various focus group studies in the field of health services research.

GÖTZ FABRY is a physician working in the Department of Medical Psychology and Sociology at Freiburg University Medical School. His current research focuses on competency based education as well as epistemological beliefs.

TOBIAS PUKROP is a fellow in the Department of Hematology and Oncology at Göttingen University Hospital. He was involved in developing the university’s medical curriculum and a novel evaluation tool. In addition, he has been running a student-led PBL course in haematology for 8 years.

SVEN ANDERS works as a consultant in the Department of Legal Medicine at Hamburg University, co-ordinating the department’s teaching activities. He is involved in curricular development and has just completed a two-year study course of Medical Education. Main research areas are forensic pathology, clinical forensic medicine, and medical education.

TOBIAS RAUPACH is a cardiologist who works in the Department of Cardiology and Pneumology at Göttingen University. He co-ordinates the department’s teaching activities and has helped to develop the institution’s curriculum. His current research focuses on curricular development, evaluation and assessment formats.

## Pre-publication history

The pre-publication history for this paper can be accessed here:

http://www.biomedcentral.com/1472-6920/12/45/prepub
